# Electric‐Field Manipulation of Magnetic Chirality in a Homo‐Ferro‐Rotational Helimagnet

**DOI:** 10.1002/advs.202402048

**Published:** 2024-07-03

**Authors:** Junjie Yang, Masaaki Matsuda, Trevor Tyson, Joshua Young, William Ratcliff, Yunpeng Gao, Dimuthu Obeysekera, Xiaoyu Guo, Rachel Owen, Liuyan Zhao, Sang‐wook Cheong

**Affiliations:** ^1^ Department of Physics New Jersey Institute of Technology Newark NJ 07102 USA; ^2^ Neutron Scattering Division Oak Ridge National Laboratory Oak Ridge TN 37831 USA; ^3^ Department of Chemical and Materials Engineering New Jersey Institute of Technology Newark NJ 07102 USA; ^4^ NIST Center for Neutron Research National Institute of Standards and Technology Gaithersburg MD 20899 USA; ^5^ Department of Materials Science and Engineering Department of Physics University of Maryland College Park MD 20741 USA; ^6^ Department of Physics University of Michigan Ann Arbor MI 48109 USA; ^7^ Rutger Center for Emergent Materials and Department of Physics and Astronomy Rutgers University Piscataway NJ 08854 USA

**Keywords:** ferro‐rotation, helimagnet, magnetic chirality, multiferroic, neutron diffraction, single crystal growth

## Abstract

Ferro‐rotational (FR) materials, renowned for their distinctive material functionalities, present challenges in the growth of homo‐FR crystals (i.e., single FR domain). This study explores a cost‐effective approach to growing homo‐FR helimagnetic RbFe(SO_4_)_2_ (RFSO) crystals by lowering the crystal growth temperature below the *T_FR_* threshold using the high‐pressure hydrothermal method. Through polarized neutron diffraction experiments, it is observed that nearly 86% of RFSO crystals consist of a homo‐FR domain. Notably, RFSO displays remarkable stability in the FR phase, with an exceptionally high *T_FR_* of ≈573 K. Furthermore, RFSO exhibits a chiral helical magnetic structure with switchable ferroelectric polarization below 4 K. Importantly, external electric fields can induce a single magnetic domain state and manipulate its magnetic chirality. The findings suggest that the search for new FR magnets with outstanding material properties should consider magnetic sulfates as promising candidates.

## Graphical abstract

This study investigates the cost‐effective growth of homo‐FR RbFe(SO_4_)_2_ crystals, showcasing their stability in the FR phase at 573 K. Neutron diffraction reveals 86% of a homo‐FR domain. Its helical magnetic structure and switchable ferroelectric polarization are explored, along with the potential for electric field‐induced chiral magnetic domain control. Promising sulfates as FR materials are highlighted.

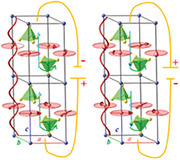

## 1 Introduction

Ferro‐rotational (FR) materials that exhibit spontaneous ordering of crystallographic rotational distortions have garnered significant attention due to their unique material functionalities.^[^ ^1^, ^2^, ^3^, ^4^, ^5^, ^6^, ^7^, ^8^, ^9^, ^10^, ^11^, ^12^ ^]^ These rotational distortions can arise from either clockwise (CW) or counterclockwise (CCW) rotations of atomic polyhedra within crystals, leading to the emergence of an axial‐vector order parameter known as Ferro‐rotation (FR) across extensive spatial dimensions.^[^ ^5^, ^13^ ^]^ When combined with the non‐collinear helical spin order, this FR order can result in multiferroicity in FR helimagnets.^[^ ^2^, ^12^ ^]^ Remarkably, multiferroicity in FR helimagnets opens up the potential to manipulate various material properties using electric fields. This includes controlling the helical spin order and spin waves, paving the way for innovative device designs.^[^ ^14^, ^15^, ^16^, ^17^, ^18^ ^]^ However, achieving the manipulation of material properties under electric fields in FR helimagnets necessitates the presence of a homo‐FR domain state (i.e., a single FR domain which is either CCW or CW). In contrast to other ferroic materials, like ferroelectric materials, where the ferroelectric domain can be easily controlled by the conjugate electric field $\overset{⇀}{E}$, the conjugate field to FR order is the curl of $\overset{⇀}{E}$, i.e., $\nabla \times \overset{⇀}{E}$, which often lacks the required intensity to govern the FR domain.^[^ ^13^ ^]^ Consequently, growing homo‐FR helimagnets presents significant challenges, and there is limited literature documenting successful attempts with known FR helimagnets.

The search for new FR helimagnets that require the growth of a homo‐FR domain is crucial for exploring the functionalities of FR materials. One approach is to seek out materials with a high FR transition temperature (*T_FR_*) and a low crystal growth temperature (*T_g_*), especially when *T_g_* < *T_FR_*. In such cases, the crystal may naturally develop into a homo‐FR domain during the crystallization process. To date, known FR magnets either have *T_g_* > *T_FR_*, as seen in RbFe(MoO_4_)_2_ (RFMO) and CaMn_7_O_12_ (CMO), resulting in a multi‐domain state, or they exhibit collinear spin order rather than the desired non‐collinear helical order, as observed in MTiO_3_ (M = Mn, Fe, and Ni).^[^ ^1^, ^2^, ^5^, ^19^, ^20^ ^]^ Sulfate‐containing magnetic ions may serve as promising candidates for achieving high *T_FR_* with magnetic order. Compared to other oxygen polyhedra, SO_4_ ions are smaller, thus possessing higher mobility features that can manifest strong rotational properties. However, very few magnetic sulfates have been reported to form bulk single crystals, primarily due to growth challenges. This limitation hinders the revelation of their exotic material functionalities. For instance, the magnetic anhydrous sulfate AM(SO_4_)_2_ (where A = K, Rb, Cs, and M = Cr or Fe) exhibits potential FR $P\bar{3}$ space group at room temperature, with magnetic ordering occurring below 4 K, as suggested by previous powder diffraction results.^[^ ^21^, ^22^, ^23^, ^24^ ^]^ However, to date, only powder samples have been available, with none successfully grown into single crystals. Consequently, the precise nature of these materials remains poorly understood.

In our current research, we have successfully obtained unique homo‐FR helimagnetic crystals of magnetic anhydrous sulfate RbFe(SO_4_)_2_ (RFSO) using a cost‐effective hydrothermal method. Our X‐ray diffraction analysis confirms that RFSO possesses an exceptionally high *T_FR_* of ≈573 K, significantly exceeding its *T_g_* of ≈483 K. Our first‐principle calculations indicate that the high *T_FR_* in RFSO is a result of the extremely high stability of the SO_4_ tetrahedra in the FR phase. Additionally, polarized neutron diffraction studies reveal that roughly 86% of the RFSO crystal consists of a homo‐FR domain. At temperatures below 4.0 K, RFSO exhibits a chiral helical spin order and a ferroelectric polarization along the *c*‐axis. An external electric field can induce a single magnetic chiral domain state and manipulate the magnetic chirality within the single domain. The availability of homo‐FR RFSO crystals with exceptional tunability of magnetic chirality by electric field opens up exciting possibilities for future device configurations, such as electric‐field tunable spin‐wave diodes. This study suggests the potential for synthesizing new homo‐FR helimagnets with unique properties and applications within SO_4_‐based magnetic materials.

## 2 Results and Discussion

**Figure 1:**
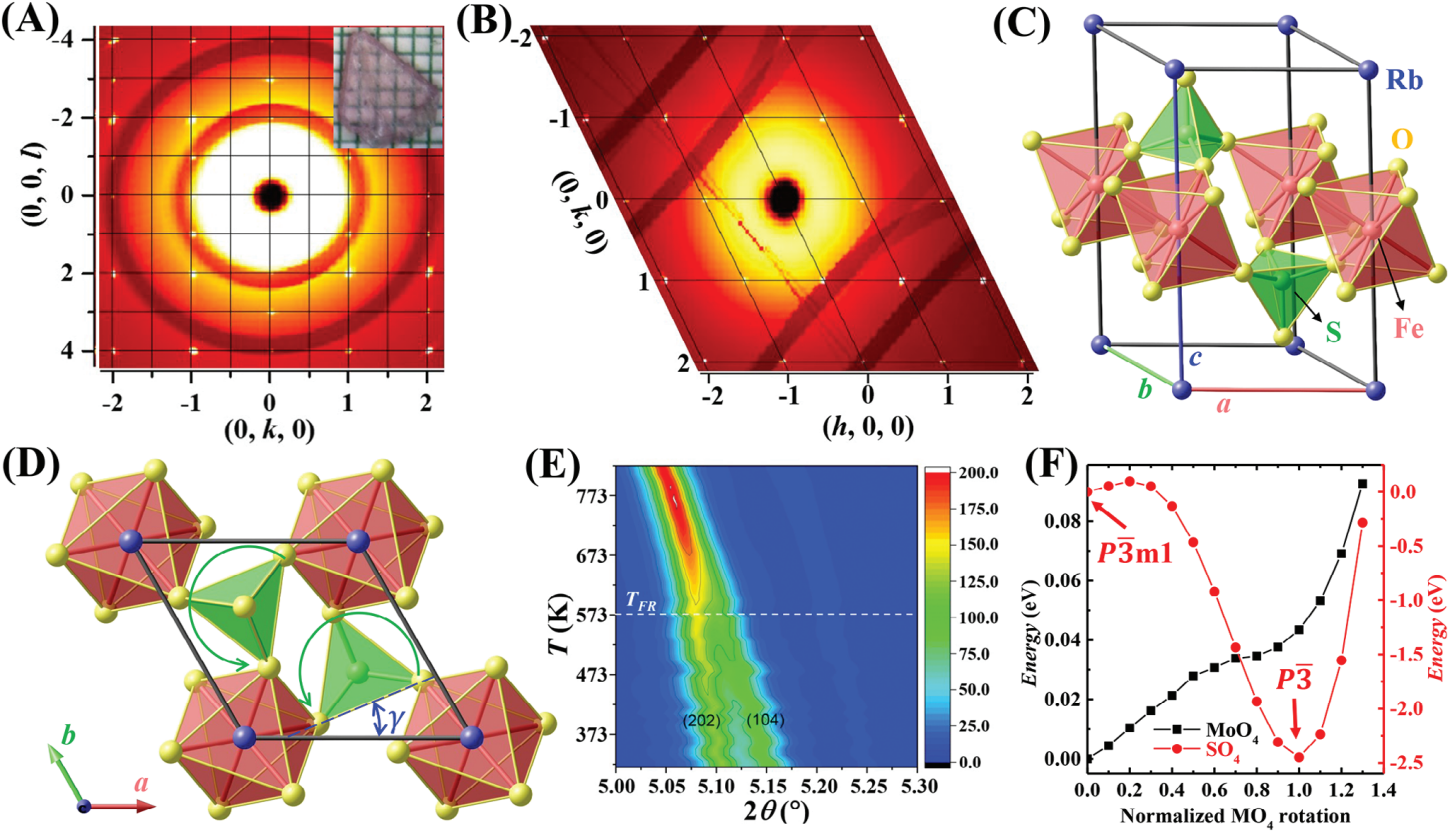
A) X‐ray single crystal diffraction pattern in the (0, *k*, *l*) plane at 290 K. The inset displays a photograph of an RFSO single crystal on a millimeter‐grid paper. B) X‐ray single crystal diffraction pattern in the (*h*, *k*, 0) plane at 290 K. C) Refined crystal structure of RFSO at 290 K. D) The CCW rotational distortions (green circles with arrows) of SO_4_ and FeO_6_ for the type‐I FR domain. The angle *γ* represents the rotational distortion relative to the *a*‐axis. These rotational distortions break mirror reflection symmetries in the *ab* plane. E) Temperature‐dependent X‐ray diffraction patterns for an RFSO powder sample. The white dashed line serves as a guide to visualize the FR phase transition. F) Calculated energy as a function of MO_4_ (M = S or Mo) polyhedral rotation angle for RFSO (red curve) and RFMO (black curve). The rotation angle is normalized to the equilibrium rotational angle identified in the optimized $P\bar{3}$ structure.

Under the condition of *T_g_* < *T_FR_*, a tiny nucleus forms at the initial stage of crystal growth, likely a homo‐FR domain due to its small size. As the crystallization progresses, this homo‐FR domain nucleus acts as a seed, absorbing free atoms and propagating its homo‐FR structure outward to form a bulk crystal. Conversely, when *T_g_* > *T_FR_*, as exemplified by RFMO with *T_FR_* ≈ 190 K and *T_g_* > 700 K and CMO with *T_FR_* ≈ 440 K and *T_g_* ≈ 923 K, once the crystal cools below *T_FR_*, both CW and CCW domains populate equally.^[^ ^2^, ^25^, ^26^ ^]^ This scenario emphasizes the importance of identifying an FR system with a high *T_FR_* and utilizing a growth method capable of significantly reducing *T_g_*. Hence, we chose RFSO for our study, where the SO_4_ polyhedra may have greater stability than MoO_4_ in the FR phase, and thus have high *T_FR_*. For crystal growth, we opted for the low‐cost hydrothermal method, which provides a high‐pressure environment, enhancing the solubility of RFSO in the solvent and thereby reducing *T_g_*. Our RFSO crystals were successfully grown at 483 K, exhibiting a hexagonal platelike morphology, as depicted in the inset of **Figure** **1**A. Detailed growth information can be found in the experimental section. To determine the precise crystal structure of RFSO, we conducted synchrotron X‐ray diffraction experiments on an RFSO crystal (#1). Figure 1A,B show the X‐ray diffraction patterns obtained at room temperature. Bright spots at the intersection of the (*h*, *k*, *l*) grids are the Bragg reflections of RFSO single crystal. The broad dark stripes are from dead zones in the area detector, separating active regions, and the sun‐like disks at the center are due to air scattering as well as scattering from epoxy holding the crystal to the support fiber. The crystal structure was refined in three different space groups: $P\bar{3}$, *P*321, and $P\bar{3}m1$, with results summarized in **Table** **1**. The methods used to analyze the single‐crystal X‐ray diffraction data and the refined parameters of the $P\bar{3}$, *P*321, and $P\bar{3}m1$ models are presented in the Supporting Information. Among these space groups, $P\bar{3}$ was determined to offer the best fit to the data (smallest *R_1_*, smallest residual, and *GOF* close to 1.0), corresponding to the FR structure. The $P\bar{3}$ structure is depicted in Figure 1C,D, and it is composed of alternating layers of SO_4_ tetrahedra, FeO_6_ octahedra, and Rb ions. As shown in Figure 1C,D, the SO_4_ tetrahedra exhibit a CCW rotation around the *c*‐axis for the type‐I FR domain, while for the type‐II FR domain, they display a CW rotation (as shown in Figure S2A,B, Supporting Information). The angle *γ*, representing the rotation of SO_4_ relative to the *a*‐axis, is ≈25°.

**Table 1:** Quality of Fit Parameters for Different Space Groups. The *R*_1_ is defined as *R*_1_ = ∑||*F_o_* | − |*F_c_* ||/∑|*F_o_* |, where *F_o_* is the measured scattering amplitude and *F_c_* is the calculated amplitude. The residual (charge) represents the Max and Min Peak in the Final Difference Map. The *GOF* (Goodness of fit) is defined as $\mathrm{GOF}={\left\{\sum \;w{\left(F_{2}^{O}-F_{2}^{c}\right)}^{2}/\left(n-p\right)\right\}}^{1/2}$, where *w* is the weight, *n* is the number of reflections, and *p* is the total number of fitting parameters.

Space Group^a^ ^)^	***R***_1_	Residual [e A^−3^]	*GOF*	Temperature [K]
$P\bar{3}$	3.90%	2.12/−1.26	1.076	290
*P*321	8.65%	3.29/−2.06	1.164	290
$P\bar{3}m1$	11.50%	4.93/−2.06	1.266	290

^a)^ Refined Rb occupancy is 0.870(4) for $P\bar{3}$, 0.849(15) for *P*321, 1.02(2) for $P\bar{3}m1$.

To determine the *T_FR_* of RFSO, high‐temperature synchrotron powder X‐ray diffraction measurements were conducted on an RFSO powder sample (crushed crystals). Figure 1E shows the contour plot of diffraction patterns ranging from 323 to 823 K. A continuous transition is observed near 573 K, indicated by the merging of (202) and (104) peaks of the $P\bar{3}$ structure, suggesting a *T_FR_* ≈ 573 K which is significantly higher than that of RFMO (*T_FR_* ≈ 190 K). To gain a better understanding of the high *T_FR_* in RFSO, we conducted Density Functional Theory (DFT) calculations, comparing it with its counterpart RFMO. First, we fully optimized the crystal structures of both RFSO and RFMO in both the FR $P\bar{3}$ and non‐FR $P\bar{3}m1$ phases (see Figure S2C–F, Supporting Information). Subsequently, we calculated the energy as a function of the rotational angle of MO_4_ (M = S or Mo), as illustrated in Figure 1F. In Figure 1F, the rotational angles are normalized to that of the optimized $P\bar{3}$ phase for each material. In the case of RFSO, the energy‐rotation curve displays a deep valley near the optimized $P\bar{3}$ phase (i.e., where the normalized SO_4_ rotation equals to 1), with an energy gain of 2.5 eV induced by the SO_4_ rotation. A slight deviation from the optimized $P\bar{3}$ phase significantly decreases the energy gain. In contrast, the energy‐rotation curve of RFMO is nearly flat, and MoO_4_ rotation does not result in a significant energy gain. These results suggest that the rotational distortion of SO_4_ in RFSO is highly stable, and the high *T_FR_* observed in RFSO is attributed to the stability of SO_4_ rotation, which is significantly more energetically unfavorable to turn off (leading to the non‐FR $P\bar{3}m1$ phase) compared to MoO_4_ rotations.

**Figure 2:**
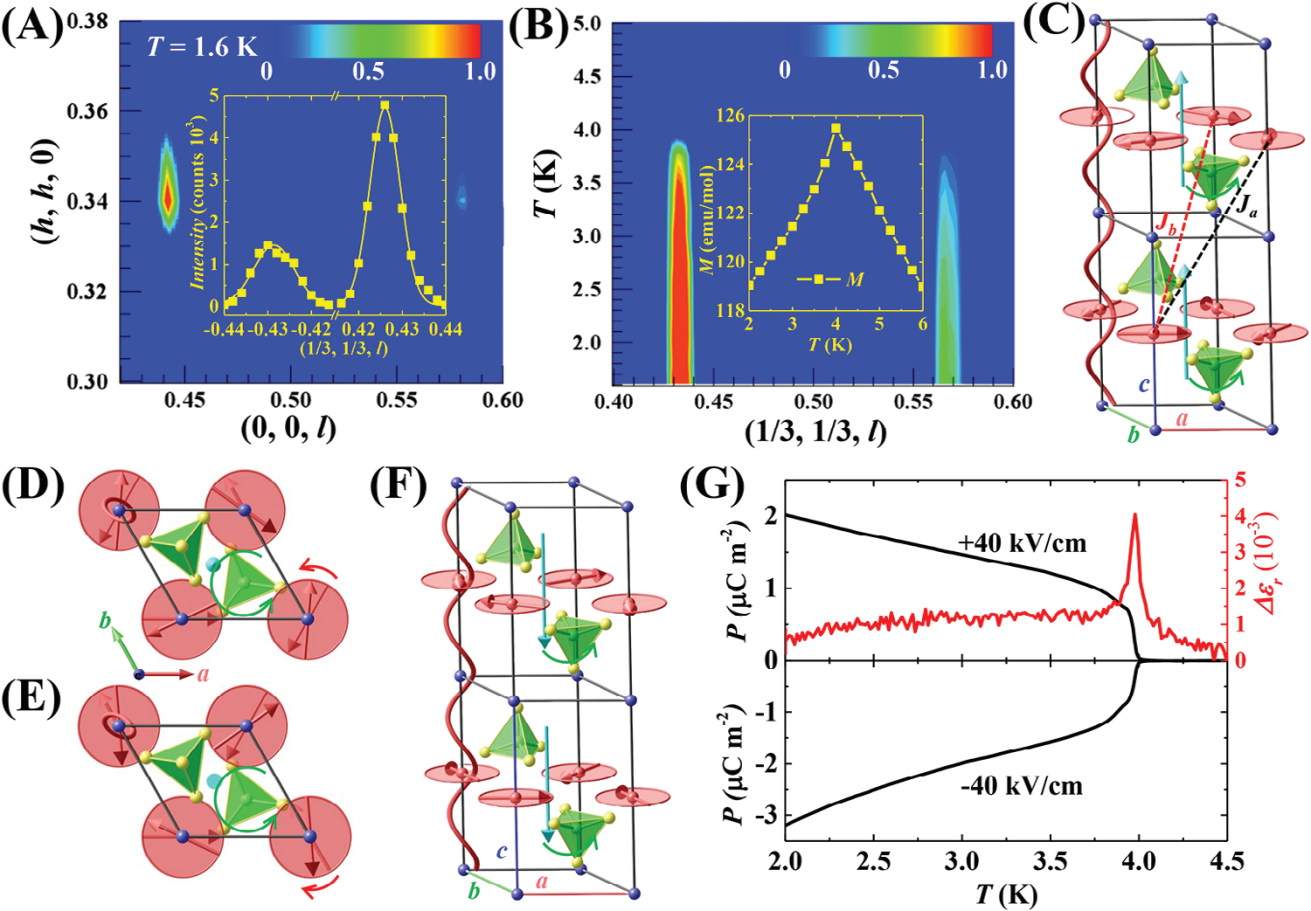
A) Unpolarized neutron diffraction pattern in the (*h*, *h*, *l*) scattering plane measured at 1.6 K on RFSO single crystal (#2). The inset provides a comparison of neutron diffraction intensity at (1/3, 1/3, –0.43) and (1/3, 1/3, +0.43) magnetic peaks. Error bars in this paper represent one standard deviation. B) Temperature dependence of the (1/3, 1/3, 0.43) and (1/3, 1/3, 0.57) magnetic peaks. The inset displays the temperature dependence of the magnetic moment for an RFSO single crystal when a magnetic field is applied to the *ab* plane with a magnitude of 0.2 T. C,D) A CCW FR domain with a right‐handed helical magnetic structure exhibits upward electric polarization (light blue arrow). The green circle arrow in represents the FR order, and the red circle arrow in illustrates the twist of magnetic moments in the adjacent layers. The black dashed line in (C) indicates the super‐exchange path *J_a_*, while the red dashed line illustrates *J_b_*. E,F) A CCW FR domain with a left‐handed helical magnetic structure exhibits downward electric polarization (light blue arrow). G) Electric polarization *P_c_* as a function of temperature (black left axis). The right red axis exhibits the relative change of dielectric constant as a function of temperature, represented as Δ*ε* = *ε*(*T*)*‐ε*(5 K).

The RFSO crystals grown under the condition of *T_g_* < *T_FR_* is likely homo‐FR. However, determining the population of FR domains within a bulk crystal presents challenges. Thanks to the interactions between FR and magnetic order, an alternative approach to utilizing magnetic peaks by the neutron diffraction method is feasible. Unpolarized neutron diffraction experiments were conducted on an RFSO crystal (#2, ≈5 × 5 × 1 mm^3^). **Figure** **2**A displays the neutron diffraction patterns in the (*h*, *h*, *l*) plane at 1.6 K. A pair of incommensurate magnetic peaks were observed at (1/3, 1/3, 0.5 ± Δ) with Δ ≈0.07. Our representational analysis, outlined in detail in the Supporting Information, of the magnetic peaks indicates the possibility of three magnetic structures: Γ_1_, Γ_2_, and Γ_3_.^[^ ^27^, ^28^, ^29^, ^30^ ^]^ Γ_1_ corresponds to a spin‐density wave structure (see Figure S3A, Supporting Information) with magnetic moments aligned along the *c*‐axis, which is non‐chiral. On the other hand, Γ_2_ and Γ_3_ represent left‐handed and right‐handed chiral helical magnetic structures (see Figure S3B,C, Supporting Information), respectively. As discussed later in this manuscript, our polarized neutron diffraction results revealed the magnetic chirality in RFSO, decisively excluding the possibility of the Γ_1_ structure. Either Γ_2_ or Γ_3_ yields a chiral magnetic structure with a 120° configuration within each layer and a chiral helical twist between adjacent layers along the *c*‐axis. Figure 2C–F shows the chiral helical magnetic structure of RFSO at 1.6 K. It's worth noting that another pair of incommensurate magnetic peaks were also observed at (2/3, 2/3, 0.5 ± Δ) (Figure S4A, Supporting Information). Figure 2B displays the temperature dependence of the (1/3, 1/3, 0.5 ± Δ) peaks, with peak intensities increasing below 4 K, suggesting a magnetic ordering temperature of 4 K. This is consistent with the results of the temperature‐dependent magnetic moment (*M*–*T*) shown in the inset of Figure 2B. The *M*–*T* curve exhibits a distinct peak at 4 K, indicating an antiferromagnetic transition at *T_N_* ≈ 4 K.

The chiral helical magnetic structure in RFSO may arise from its FR order. The FR order breaks mirror reflection symmetries in the *ab* plane of RFSO, resulting in nonequivalent super‐super‐exchange terms *J_a_* (dashed black) and *J_b_* (dashed red) bridging adjacent triangular lattice layers, as illustrated in Figure 2C. These nonequivalent *J_a_* and *J_b_* terms induce a relative twist between adjacent layers, leading to the chiral helical magnetic structure. The two distinct FR domains have different values of *J_a_* and *J_a_*: if *J_a_* is smaller than *J_a_* in the CW domain, then it should be the case that *J_a_* is larger than *J_b_* in the CCW domain.^[^ ^1^ ^]^ Consequently, the two FR domains exhibit distinct magnetic propagation vectors, denoted as ${\overset{⇀}{q}}_{1}=\;\left(\pm 1/3,\pm 1/3,\pm q_{z}\right)$ for CW domain, and ${\overset{⇀}{q}}_{2}=\;\left(\mp 1/3,\mp 1/3,\pm q_{z}\right)$ for the CCW domain.^[^ ^1^ ^]^ In a crystal with an even distribution of FR domains, the magnetic peaks associated with ${\overset{⇀}{q}}_{1}$ should exhibit comparable intensity to those of ${\overset{⇀}{q}}_{2}$. However, as Figure 2A illustrates, the intensity of (1/3, 1/3, 0.43) significantly exceeds that of (1/3, 1/3, 0.57), indicating the dominance of one FR domain. This observation holds true for (2/3, 2/3, 0.5 ± Δ) (see Figure S4A, Supporting Information). As shown in the inset of Figure 2A, a fitting analysis on the (1/3, 1/3, 0.43) and (1/3, 1/3, −0.43) peaks reveals an integrated peak area ratio of 3.3:1, suggesting that the dominant FR domain constitutes ≈77% of the crystal. To further confirm the homo‐FR state, we conducted Rotational‐Anisotropy Second Harmonic Generation measurements at four positions on two different crystals (#3 and #4), indicating a homo‐FR domain in RFSO crystals extending at least 2 mm (see Figure S5, Supporting Information), ≈50 times larger than that of RFMO (≈40 µm).^[^ ^5^ ^]^

To uncover the potential novel material functionalities in the (almost) homo‐FR RFSO crystals, we have harnessed the concept of permutable Symmetry Operational Similarity (SOS) which has proven highly effective in comprehending material properties.^[^ ^13^, ^31^, ^32^, ^33^, ^34^ ^]^ One key permutable SOS relationship is expressed as **A•C = P**.^[^ ^34^ ^]^ Here, **A** represents the FR vector, **C** is chirality, and **P** corresponds to electric polarization. This relationship informs us that when an FR object **A** interacts with chirality **C**, the resultant **A•C** response manifests as an electric polarization **P**. The chiral helical magnetic structure observed in RFSO exhibits characteristics reminiscent of chirality **C**. Consequently, RFSO possesses ferroelectricity, and the direction of its ferroelectric polarization depends on the sense of FR and the chiral helical magnetic structure. We assume that the dominant FR domain in RFSO is of the CCW type. As visually depicted in Figure 2C–F, within a CCW FR system, a right‐handed (left‐handed) chiral helical magnetic structure induces an upward (downward) electric polarization along the *c*‐axis. Conversely, within a CW FR system, a left‐handed (right‐handed) chiral helical magnetic structure results in an upward (downward) electric polarization. Furthermore, it's important to note that the SOS relationship **A•C = P** is permutable, meaning that **A•P = C** is also valid. This implies that the chirality of the helical magnetic structure can be controlled by the electric polarization. As illustrated in Figure 2C–F, inverting the polarization direction could switch the magnetic chirality in a homo‐FR crystal.

We measured the electric polarization along the *c‐*axis (*P_c_*) of an RFSO crystal (#5) as a function of temperature (*P_c_*–*T*). Before the measurement, the crystal was poled by an external electric field during the cooling process. Figure 2G exhibits the *P_c_*–*T* curve obtained under a positive poling field +40 kV cm^−1^. In line with the onset of helical magnetic order, *P_c_* begins to increase at 4 K and reaches a value of ≈2.0 µC m^−2^ at 2 K. These observations provide strong evidence supporting the idea that the combination of FR and chiral helical magnetic structures can induce ferroelectric polarization. The temperature dependence of the dielectric constant ε was also measured. Figure 2G (on its right axis, shown in red) exhibits the relative change of dielectric constant as a function of temperature, represented as Δ*ε* = *ε*(*T*)*‐ε*(5 K). A notable peak is observed at *T_N_* ≈4 K in the Δ*ε–T* curve. To examine the switchability of *P_c_*, the *P_c_*–*T* curve was also measured under a negative poling field of −40 kV cm^−1^. As shown in the bottom panel of Figure 2G, the polarization *P_c_* is symmetric and switchable.

**Figure 3:**
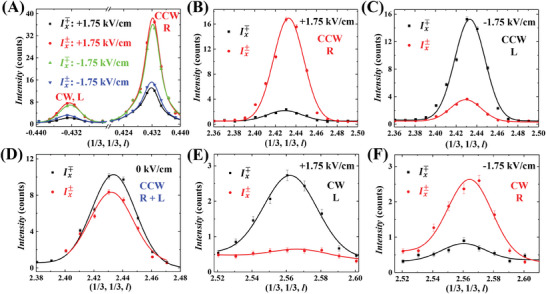
A) Intensities of $I_{x}^{\pm }$ and $I_{x}^{\mp }$ measured at (1/3, 1/3, –0.43) and (1/3, 1/3, 0.43) magnetic peaks with two different electric poling fields. B) Intensities of $I_{x}^{\pm }$ and $I_{x}^{\mp }$ measured at (1/3, 1/3, 2.43) with a +1.75 kV cm^−1^ poling field. C) Intensities of $I_{x}^{\pm }$ and $I_{x}^{\mp }$ measured at (1/3, 1/3, 2.43) with a –1.75 kV cm^−1^ poling field. D) Intensities of $I_{x}^{\pm }$ and $I_{x}^{\mp }$ measured at (1/3, 1/3, 2.43) with 0 kV cm^−1^ poling field. E) Intensities of $I_{x}^{\pm }$ and $I_{x}^{\mp }$ measured at (1/3, 1/3, 2.56) with +1.75 kV cm^−1^ poling field. F) Intensities of $I_{x}^{\pm }$ and $I_{x}^{\mp }$ measured at (1/3, 1/3, 2.56) with a –1.75 kV cm^−1^ poling field. All results were measured at 1.6 K. The CCW and CW letters indicate the type of FR domain, and the R and L letters signify the handedness of the magnetic domain.

To ascertain whether the chirality of helical magnetic structure can be switched by an electric field, we performed the polarized neutron diffraction experiments on an RFSO crystal (#6, ≈3 × 3 × 1 mm^3^). We define the *x*‐axis along the direction of the scattering vector $\overset{⇀}{Q}$, with *z* being vertical and *y* perpendicular to *x* in the scattering plane. The incident neutrons were polarized along the *x*‐axis (i.e., ${\overset{⇀}{P}}_{i}∥\overset{⇀}{Q}$) and the analysis was performed in the same direction. With two possible polarization states for both incident and scattered neutrons (either parallel or antiparallel to *x*), we can measure four distinct intensities: $I_{x}^{++}$, $I_{x}^{--}$, $I_{x}^{\pm }$, and $I_{x}^{\mp }$. The $I_{x}^{\pm }\propto {|M_{⊥}|}^{2}+P_{i}M_{C}$ and $I_{x}^{\mp }\propto {|M_{⊥}|}^{2}-P_{i}M_{C}$, where *M*_⊥_ is the magnetic component perpendicular to $\overset{⇀}{Q}$ and *M_C_* is the magnetic chiral term with $M_{C}=\;i\left(M_{⊥}^{y*}M_{⊥}^{z}-M_{⊥}^{z*}M_{⊥}^{y}\right)$. For an effective comparison of the measured magnetic chirality at different magnetic peaks, we introduce a normalized magnetic chirality $m_{C}=\;\left(I_{x}^{\pm }-I_{x}^{\mp }\right)/\left(I_{x}^{\pm }+I_{x}^{\mp }\right)$. The crystal was initially poled under +1.75 kV cm^−1^ upon cooling. Given the poling process, each FR domain would coincide with a single ferroelectric domain and a single magnetic helical domain. To deduce the proportion of the two FR domains, we compared the intensity of $I_{x}^{\pm }$ (or $I_{x}^{\mp }$) for the two (1/3, 1/3, ± 0.43) peaks, corresponding to the two distinct FR domains. Integrated intensities of these peaks were computed from their fittings, as shown in **Figure** **3**A. For +1.75 kV cm^−1^ (red curves), the integrated intensities of $I_{x}^{\pm }$ at (1/3, 1/3, 0.43) is ≈6.2 times larger than that of (1/3, 1/3, –0.43), suggesting a ratio of 86% for the dominant FR domain in crystal #6. These results indicate crystal #6 is largely a homo‐FR crystal, with the dominant FR domain assumed to be CCW. Moreover, the prominence of $I_{x}^{\pm }$ over $I_{x}^{\mp }$ at (1/3, 1/3, 0.43) suggests a right‐handed magnetic chirality in the dominant CCW FR domain for +1.75 kV cm^−1^, with an estimated *m_C_* of +0.46 based on the integrated intensities. For (1/3, 1/3, –0.43) peak, the $I_{x}^{\pm }$ is also stronger than $I_{x}^{\mp }$. However, since the scattering vector *q_z_* is negative, the magnetic domain associated with the minor CW FR domain is left‐handed, leading to a negative *m_C_*  ≈ –0.62. When the crystal is poled under –1.75 kV cm^−1^, $I_{x}^{\mp }$ dominates over $I_{x}^{\pm }$ at (1/3, 1/3, 0.43). This highlights the switchable magnetic chirality under the electric field for both CCW and CW FR domains. For –1.75 kV cm^−1^, the estimated *m_C_* is –0.42 for (1/3, 1/3, 0.43).

According to the previous results of the isostructural RFMO, the helical magnetic structure of our RFSO crystals may consist of two types of magnetic chirality: triangular and helical.^[^ ^1^ ^]^ Nevertheless, the SOS relationships suggest that the FR order and ferroelectric polarization are coupled to the helical chirality. Note that the helical chirality in RFSO arises from the twist along the (001) direction, while the triangular chirality is a result of the in‐plane 120° configuration. By analyzing the angle dependence of magnetic chirality between the (001) and (110) directions, we can differentiate the couplings of the FR order and ferroelectric polarization to each of the chirality. Figure 3B shows the $I_{x}^{\mp }$ and $I_{x}^{\pm }$ at (1/3, 1/3, 2.43) for the crystal poled with +1.75 kV cm^−1^. The scattering vector (1/3, 1/3, 2.43) forms an angle of 24.877° with the (001) direction, while the (1/3, 1/3, 0.43) has an angle of 69.023° with the same direction. Notably, the difference between $I_{x}^{\mp }$ and $I_{x}^{\pm }$ at (1/3, 1/3, 2.43) peak significantly exceeds that of the (1/3, 1/3, 0.43) peak. The estimated *m_C_* is +0.92 for (1/3, 1/3, 2.43), a value noticeably larger than that of the (1/3, 1/3, 0.43) peak. Figure 3C shows the $I_{x}^{\mp }$ and $I_{x}^{\pm }$ for (1/3, 1/3, 2.43) when the crystal is poled by –1.75 kV cm^−1^. Similar to the (1/3, 1/3, 0.43) peak, the negative electric field inverts the handedness of the magnetic chirality. The estimated *m_C_* is –0.64, again magnitude larger than that of the (1/3, 1/3, 0.43) peak. When the crystal remains unpoled (0 kV cm^−1^) as shown in Figure 3D, the $I_{x}^{\mp }$ and $I_{x}^{\pm }$ showcase nearly identical intensities, indicating the roughly equal distribution of left‐and right‐handed magnetic domains. For the minor CW FR domain, as shown in Figure 3E,F, the handedness of the magnetic chirality at (1/3, 1/3, 2.56) aligns with that of the (1/3, 1/3, −0.43) peak, albeit with a considerably elevated *m_C_*. The *m_C_* values are –0.81 and +0.63 for +1.75 and −1.75 kV cm^−1^, respectively.

**Figure 4:**
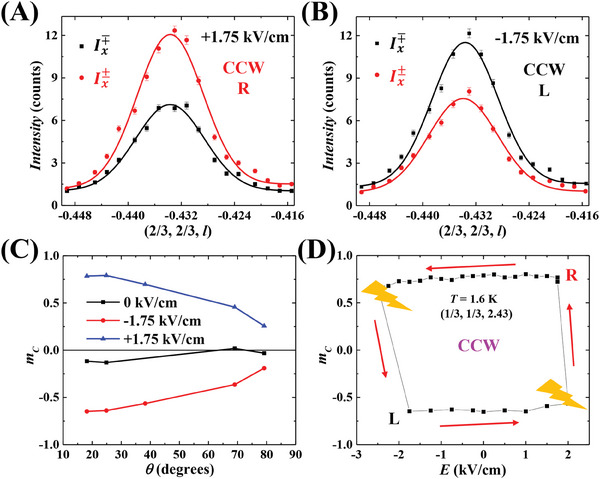
A) Intensities of $I_{x}^{\pm }$ and $I_{x}^{\mp }$ measured at (2/3, 2/3, –0.43) with a +1.75 kV cm^−1^ poling field. B) Intensities of $I_{x}^{\pm }$ and $I_{x}^{\mp }$ measured at (2/3, 2/3, –0.43) with a –1.75 kV cm^−1^ poling field. C) The angle dependence of the normalized magnetic chirality *m_c_*. The angle *θ* is the respect to (001) direction. The results were obtained at 1.6 K. D) Electric field dependence of the normalized magnetic chirality *m_c_*. The data were measured at 1.6 K at (1/3, 1/3, 2.43) magnetic peak. The red arrows illustrate the ramping direction of electric fields. All results were measured at 1.6 K. The CCW and CW letters indicate the type of FR domain, and the R and L letters signify the handedness of the magnetic domain.

**Figure** **4**A,B show the $I_{x}^{\mp }$ and $I_{x}^{\pm }$ for (2/3, 2/3, −0.43) under various poling electric fields. Note that (2/3, 2/3, −0.43) can be represented as (1, 1, 0) – (1/3, 1/3, 0.43), signifying its origin from the dominant CCW FR domain. However, when compared to (1/3, 1/3, 0.43) and (1/3, 1/3, 2.43), the (2/3, 2/3, −0.43) is proximal to the (110) direction given its minimal angle of 10.841° with it. Intriguingly, for both ±1.75 kV cm^−1^, the difference between $I_{x}^{\mp }$ and $I_{x}^{\pm }$ is notably reduced compared to that of the (1/3, 1/3, 0.43) and (1/3, 1/3, 2.43) peaks. The estimated *m_C_* are +0.26 and –0.19 for +1.75 and –1.75 kV cm^−1^, respectively. If the crystal is unpoled (see Figure S4B, Supporting Information), the $I_{x}^{\mp }$ is almost identical with $I_{x}^{\pm }$. We also measured the $I_{x}^{\mp }$ and $I_{x}^{\pm }$ at magnetic peaks of (1/3, 1/3, 1.43) and (1/3, 1/3, 3.43). The *m_C_* values for all magnetic peaks are summarized in Figure 4C, plotted against the angle *θ* relative to the (001) direction. For both +1.75 and –1.75 kV cm^−1^, the *m_C_* value decreases with increasing *θ*. This trend aligns with the SOS findings, reinforcing the coupling of FR order and ferroelectric polarization with helical rather than triangular chirality.

To assess the ferroelectric memory effect on magnetic chirality, we measured the *m_C_* at (1/3, 1/3, 2.43) peak at 1.6 K as a function of an electric field. Before the measurement, the crystal was poled with +1.75 kV cm^−1^. Figure 4D reveals that the *m_C_* remains constant even as the electric field transitions from a non‐zero value to zero. It retains both its sign and value, even when subjected to negative electric fields, underscoring the ferroelectric memory effect. The *m_C_* is expected to change its sign when the electric field surpasses the coercive field of ferroelectricity. Yet, the coercive field of RFSO might be considerably high, and the electric current leakage occurs at –2.5 kV cm^−1^ before reaching the coercive field. This electric current leakage produces heat, raising the crystal's temperature to ≈10 K. Post‐cooling to 1.6 K, with the crystal effectively conditioned under –1.75 kV cm^−1^, the *m_C_* reversed, settling a value of –0.64. Subsequent ramping from –1.75 to +2.0 kV cm^−1^ caused another electric current leakage at +2.0 kV cm^−1^, altering *m_C_* via the thermal cycle.

## 3 Conclusion

The research has successfully demonstrated a cost‐effective method for growing homo‐FR helimagnetic RFSO crystals by lowering the crystal growth temperature below the *T_FR_* using the high‐pressure hydrothermal method. Polarized neutron diffraction studies have revealed that ≈86% of the RFSO crystal consists of a single FR domain. This growth technique provides valuable insights that can be applied to the production of other homo‐FR materials. Furthermore, the X‐ray diffraction analysis has confirmed that RFSO exhibits an exceptionally high *T_FR_* of ≈573 K, which significantly exceeds the *T_FR_* values of RFMO and CMO. This remarkable *T_FR_* in RFSO is attributed to the presence of SO_4_ ions that promote rotational distortions. The findings suggest that the search for new FR materials with high *T_FR_* should consider sulfates as promising candidates.

Additionally, our investigation reveals that RFSO crystals exhibit chiral helimagnetic order, and their magnetic chirality can be manipulated by altering the direction of ferroelectric polarization through an external electric field. Recent theoretical calculations suggest that these helimagnets can generate nonreciprocal spin waves, which can either arise from the Dzyaloshinskii–Moriya interaction or when external magnetic fields are present.^[^ ^17^, ^35^ ^]^ The growing interest in nonreciprocal spin waves in materials is due to their potential for constructing logic devices like spin‐wave diodes.^[^ ^16^, ^36^, ^37^ ^]^ The homo‐FR helimagnetic RFSO crystals offer an ideal platform for investigating nonreciprocal spin waves in future research. Notably, the controllability of the magnetic chirality of RFSO via an external electric field opens up exciting possibilities. This controllability makes homo‐FR RFSO crystals a promising candidate for harnessing nonreciprocal spin waves through electric fields and facilitating the development of future device configurations, such as electric‐field‐tunable spin‐wave diodes. This study not only presents a reliable method for growing homo‐FR crystals but also underscores the potential for synthesizing new homo‐FR materials with unique properties and applications.

## 4 Experimental Section

Single crystals of RFSO were synthesized using a hydrothermal method in a 50‐mL Teflon‐lined autoclave. A sulfuric acid solution containing Rb_2_SO_4_ and Fe_2_(SO_4_)_3_ in a 1:1 molar ratio was subjected to hydrothermal treatment at 483 K for 72 h. After the growth process, hexagonal crystals with a ruby color were extracted from the solution. Single crystal X‐ray diffraction was conducted at beamline 15‐ID‐D at the Advanced Photon Source, Argonne National Laboratory, with a 0.41328 Å wavelength. Modeling of multiply redundant full‐sphere single crystal diffraction data sets was performed using Olex2, including absorption corrections and anomalous scattering corrections. Powder X‐ray diffraction data were collected at Beamline 28‐ID‐1 (PDF) at the National Synchrotron Light Source (NSLS2), Brookhaven National Laboratory, using a 0.1665 Å wavelength. Measurements were conducted on a powder sample (crushed single crystals) in a quartz capillary. RA‐SHG measurements were performed using an ultrafast light source with 800 nm wavelength, 40 fs pulse duration, and 200 kHz repetition rate. An achromatic lens with a beam size of 25 µm was employed, and the intensity of the reflected SHG was measured with a photomultiplier tube. Bulk magnetization was measured using a Vibrating Sample Magnetometer. The dielectric constant was determined at 1 kHz using an LCR meter, and ferroelectric polarization was measured by the pyro‐current method with an electrometer. Unpolarized neutron diffraction was performed at the BT4 triple‐axis spectrometer (2.36 Å wavelength) at the NIST Center for Neutron Research. Polarized neutron diffraction was carried out at HB1 triple‐axis spectrometer (2.46 Å wavelength) at the High Flux Isotope Reactor, Oak Ridge National Laboratory, using Heusler alloy monochromators and analyzers.

DFT calculations were conducted using the Vienna ab initio Simulation Package, employing projector‐augmented wave pseudo‐potentials.^[^ ^38^, ^39^, ^40^ ^]^ Supercells (2 × 2 × 2) of the original 12 atom unit cells were created. The geometries of these crystal structures were fully optimized (lattice parameters and atomic positions) using a 700 eV plane wave cutoff, 3 × 3 × 2 Monkhorst–Pack *k*‐point mesh, energy convergence of 10^−6^ eV, force convergence of 10^−3^ eV Å^−1^.^[^ ^41^ ^]^ The Perdew–Burke–Ernzerhof functional revised for solids (PBEsol) was utilized^[^ ^42^ ^]^; the lattice parameters of RFSO optimized using this functional (*a* = 4.8633 Å, *c* = 8.2636 Å) were in excellent agreement with the experimental results (Table S1, Supporting Information). A Hubbard U correction of U = 4.0 eV was applied to the Fe d‐states, in accordance with the analysis done in previous works.^[^ ^43^, ^44^ ^]^ To obtain the energy as a function of the MO_4_ rotation angle, intermediate structures were created and the energy was computed self‐consistently with a convergence tolerance of 10^−8^ eV.

## Conflict of Interest

The authors declare no conflict of interest.

## Supporting information

Supporting Information

## Data Availability

The data that support the findings of this study are available from the corresponding author upon reasonable request.

## References

[1] A. J. Hearmon , F. Fabrizi , L. C. Chapon , R. D. Johnson , D. Prabhakaran , S. V. Streltsov , P. J. Brown , P. G. Radaelli , Phys. Rev. Lett. 2012, 108, 237201. 23003983 10.1103/PhysRevLett.108.237201

[2] R. D. Johnson , L. C. Chapon , D. D. Khalyavin , P. Manuel , P. G. Radaelli , C. Martin , Phys. Rev. Lett. 2012, 108, 067201. 22401114 10.1103/PhysRevLett.108.067201

[3] J. Hlinka , J. Privratska , P. Ondrejkovic , V. Janovec , Phys. Rev. Lett. 2016, 116, 177602. 27176540 10.1103/PhysRevLett.116.177602

[4] T. Hayashida , Y. Uemura , K. Kimura , S. Matsuoka , D. Morikawa , S. Hirose , K. Tsuda , T. Hasegawa , T. Kimura , Nat. Commun. 2020, 11, 4582. 32917897 10.1038/s41467-020-18408-6 PMC7486364

[5] W. Jin , E. Drueke , S. Li , A. Admasu , R. Owen , M. Day , K. Sun , S. W. Cheong , L. Zhao , Nat. Phys. 2020, 16, 42.

[6] T. Hayashida , Y. Uemura , K. Kimura , S. Matsuoka , M. Hagihala , S. Hirose , H. Morioka , T. Hasegawa , T. Kimura , Phys. Rev. Mater. 2021, 5, 124409.

[7] X. Luo , D. Obeysekera , C. Won , S. H. Sung , N. Schnitzer , R. Hovden , S. W. Cheong , J. Yang , K. Sun , L. Zhao , Phys. Rev. Lett. 2021, 127, 126401. 34597104 10.1103/PhysRevLett.127.126401

[8] R. Owen , E. Drueke , C. Albunio , A. Kaczmarek , W. Jin , D. Obeysekera , S. W. Cheong , J. Yang , S. Cundiff , L. Zhao , Phys. Rev. B 2021, 103, 054104.

[9] X. Guo , R. Owen , A. Kaczmarek , X. Fang , C. De , Y. Ahn , W. Hu , N. Agarwal , S. H. Sung , R. Hovden , S. W. Cheong , L. Zhao , Phys. Rev. B 2023, 107, L180102.

[10] W. Jin , Nat. Nanotechnol. 2023, 18, 840. 37337058 10.1038/s41565-023-01414-2

[11] G. Liu , T. Qiu , K. He , Y. Liu , D. Lin , Z. Ma , Z. Huang , W. Tang , J. Xu , K. Watanabe , T. Taniguchi , L. Gao , J. Wen , J. M. Liu , B. Yan , X. Xi , Nat. Nanotechnol. 2023, 18, 854. 37169899 10.1038/s41565-023-01403-5

[12] J. S. White , C. Niedermayer , G. Gasparovic , C. Broholm , J. M. S. Park , A. Y. Shapiro , L. A. Demianets , M. Kenzelmann , Phys. Rev. B. 2013, 88, 060409.

[13] S. W. Cheong , D. Talbayev , V. Kiryukhin , A. Saxena , npj Quantum Mater. 2018, 3, 19.

[14] D. I. Khomskii , J. Magn. Magn. Mater. 2006, 306, 1.

[15] M. Bibes , A. Barthélémy , Nat. Mater. 2008, 7, 425. 18497843 10.1038/nmat2189

[16] M. Jamali , J. H. Kwon , S. M. Seo , K. J. Lee , H. Yang , Sci. Rep. 2013, 3, 3160. 24196318 10.1038/srep03160 PMC3819604

[17] F. J. Dos Santos , M. Dos Santos Dias , S. Lounis , Phys. Rev. B. 2020, 102, 104401.

[18] N. Ogawa , L. Köhler , M. Garst , S. Toyoda , S. Seki , Y. Tokura , Proc. Natl. Acad. Sci. 2021, 118, 2022927118. 10.1073/pnas.2022927118 PMC7923575 33608462

[19] G. Shirane , S. J. Pickart , Y. Ishikawa , J. Phys. Soc. Jpn. 1959, 14, 1352.

[20] G. Shirane , S. J. Pickart , R. Nathans , Y. Ishikawa , J. Phys. Chem. Solids. 1959, 10, 35.

[21] D. V. West , Q. Huang , H. W. Zandbergen , T. M. McQueen , R. J. Cava , J. Solid State Chem. 2008, 181, 2768.

[22] H. Serrano‐González , S. T. Bramwell , K. D. M. Harris , B. M. Kariuki , L. Nixon , I. P. Parkin , C. Ritter , Phys. Rev. B 1999, 59, 14451.

[23] T. Inami , J. Solid State Chem. 2007, 180, 2075.

[24] H. Serrano‐González , S. T. Bramwell , K. D. M. Harris , B. M. Kariuki , L. Nixon , I. P. Parkin , C. Ritterm , J. Appl. Phys. 1998, 83, 6314.

[25] P. V. Klevtsov , R. F. Klevtsova , J. Struct. Chem. 1977, 18, 339.

[26] S. A. Klimin , M. N. Popova , B. N. Mavrin , P. H. M. M. van Loosdrecht , L. E. Svistov , A. I. Smirnov , L. A. Prozorova , H. K. von Nidda , Z. Seidov , A. Loidl , A. Y. Shapiro , Phys. Rev. B. 2003, 68, 174408.

[27] A. S. Wills , Phys. Rev. B. 2001, 63, 64430.

[28] A. S. Wills , Phys. B 2000, 276, 680.

[29] E. F. Bertaut , Acta Crystallogr. A 1968, 24, 217.

[30] E. F. Bertaut , J. Magn. Magn. Mater. 1981, 24, 267.

[31] S.‐W. Cheong , X. Xu , npj Quantum Mater. 2022, 7, 40.

[32] S. W. Cheong , npj Quantum Mater. 2019, 4, 53.

[33] S. W. Cheong , F. T. Huang , M. Kim , Rep. Prog. Phys. 2022, 85, 124501. 10.1088/1361-6633/ac97aa 36198263

[34] S. W. Cheong , S. Lim , K. Du , F. T. Huang , npj Quantum Mater. 2021, 6, 58.

[35] S. Cheon , H. W. Lee , S. W. Cheong , Phys. Rev. B 2018, 98, 184405.

[36] D. Seo , S. Hwang , B. Kim , Y. Yang , S. Yoon , B. K. Cho , Sci. Rep. 2021, 11, 24385. 34934064 10.1038/s41598-021-02967-9 PMC8692326

[37] Q. Wang , A. V. Chumak , P. Pirro , Nat. Commun. 2021, 12, 2636. 33976137 10.1038/s41467-021-22897-4 PMC8113576

[38] P. Hohenberg , W. Kohn , Phys. Rev. 1964, 136, B864.

[39] G. Kresse , J. Furthmüller , Phys. Rev. B. 1996, 54, 11169. 10.1103/physrevb.54.11169 9984901

[40] P. E. Blöchl , Phys. Rev. B 1994, 50, 17953. 10.1103/physrevb.50.17953 9976227

[41] H. J. Monkhorst , J. D. Pack , Phys. Rev. B 1976, 13, 5188.

[42] J. P. Perdew , A. Ruzsinszky , G. I. Csonka , O. A. Vydrov , G. E. Scuseria , L. A. Constantin , X. Zhou , K. Burke , Phys. Rev. Lett. 2008, 100, 136406. 18517979 10.1103/PhysRevLett.100.136406

[43] J. Hubbard , Proc. Roy. Soc. Lond. A 1963, 276, 238.

[44] K. Cao , R. D. Johnson , F. Giustino , P. G. Radaelli , G. C. Guo , L. He , Phys. Rev. B 2014, 90, 024402.

