# Supplementary material for: Electric‐Field Manipulation of Magnetic Chirality in a Homo‐Ferro‐Rotational Helimagnet
Source: Adv Sci (Weinh). 2024 Jul 3;11(33):2402048. doi: 10.1002/advs.202402048 (PMC11434034; doi:10.1002/advs.202402048)
Supplement: Supplementary file 1 — Supporting Information [file ADVS-11-2402048-s001.docx]

Supporting Information

Electric-field Manipulation of Magnetic Chirality in a Homo-Ferro-Rotational Helimagnet

Junjie Yang*, Masaaki Matsuda, Trevor Tyson, Joshua Young, William Ratcliff, Yunpeng Gao, Dimuthu Obeysekera, Xiaoyu Guo, Rachel Owen, Liuyan Zhao, and Sang-wook Cheong

1. X-ray single crystal diffraction patterns

The following two figures depict the X-ray diffraction patterns of RbFe(SO_4_)_2_ (RFSO) single crystals obtained at room temperature. These patterns represent the same dataset as illustrated in Figure 1(A) and (B) in the main text. However, due to space limitations in the main text, we only displayed the diffraction patterns within the range from (0, 0, -4) to (0, 0, 4), from (0, -2, 0) to (0, 2, 0), and from (-2, 0, 0) to (2, 0, 0). Here, we present the data over a broader range in reciprocal space. The (*h*, *k*, *l*) grid corresponds to the $P\bar{3}$ space group. In the images, the dark stripes, indicated by the blue arrow, originate from dead zones in the area detector, which separate active regions. The sample undergoes a 360º rotation in steps of 0.3º, capturing all Bragg peaks accessible by the 2*θ* angles. The sun-like disk, indicated by the green arrow, results from air scattering as well as scattering from epoxy used to hold the crystal to the support fiber. Bright points at the intersection of the lines represent the Bragg reflections of the single crystal.


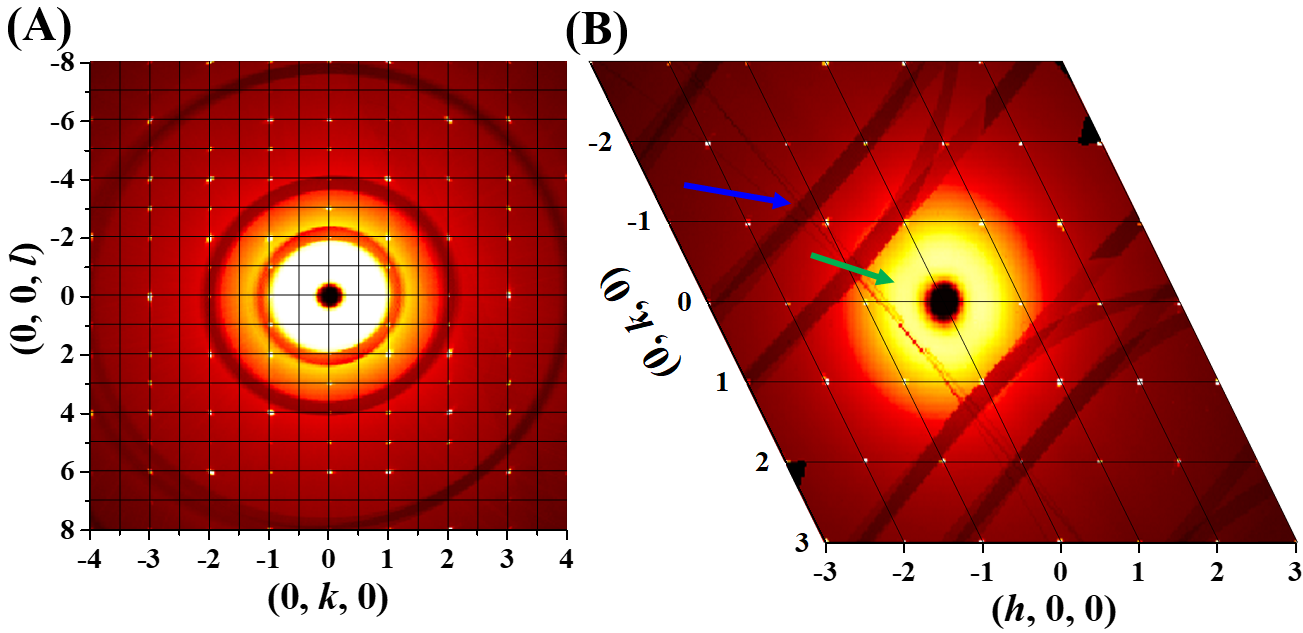


**Figure S1.** RbFe(SO_4_)_2_ single-crystal X-ray diffraction patterns of the (0, *k*, *l*) plane **(A)** and the (*h*, *k*, 0) plane **(B)**. The (*h*, *k*, *l*) grid corresponds to the hexagonal $P\bar{3}$ space group. In the images, the dark stripes, indicated by the blue arrow, stem from dead zones in the area detector, separating active regions. The sun-like disk, indicated by the green arrow, results from air scattering as well as scattering from the epoxy holding the crystal to the support fiber. Bright points at the intersection of the lines represent the Bragg reflections. Note that these images show larger regions of reciprocal space (higher (*h*, *k*, *l*) than the corresponding figures in Figure 1(A) and (B) in the main text. They represent just two planes in reciprocal space. More than 1700 reflections were found by examining complete spheres in reciprocal space.

**2. Crystal Structure Solution**

Diffraction measurements were conducted on a 50 μm × 50 μm × 5 μm single crystal at the Advanced Photon Source (APS) beamline 15-ID-D at Argonne National Laboratory using a wavelength of 0.41328 Å (30 keV). The data were collected with a PILATUS 1M CdTe detector (by DECTRIS, maximum count rate = 10^7^ cps/pixel, counter depth = 20 bit). The detector elements have a pixel size of 172 mm × 172 mm. A cryostat (Oxford) system was used to cool the sample in cold nitrogen vapor. The sample was mounted at the tip of a glass fiber with epoxy and rotated by 360º in steps of 0.3º, yielding 1200 images. The distance from the samples to the detector was 120 mm. The image data were processed using APEX3 (Bruker, 2016).^[1]^ The experimental reciprocal space precession images were generated using the same software.

The solution and refinement of the data were done using the program Olex2 after the reflections were corrected for absorption using SADABS.^[2]^ Anomalous scattering corrections (*f*’ and *f*”) were induced for all atoms. The values of *f*’ and *f*” values for Rb, Fe, S, and O at a wavelength of 0.41328 Å are 0.262 and 1.184, 0.199 and 0.306, 0.044 and 0.041 and 0.002 and 0.002, respectively. The attenuation length of the X-ray beam with *λ* = 0.41328 Å is $\approx$450 mm, indicating very low decay of the X-ray beam on transmission through the sample. No symmetry assumptions were made on the symmetry in the data collection process. Full spheres in reciprocal space were collected. This resulted in more than 3 times as many reflections with amplitude |*F*(*hkl*)| as needed for a complete data set based on symmetry. (See the ratio of measured reflections/independent reflections in the tables below). From images, $\approx$1700 reflections were harvested and used to determine the structure.

Systematic exploration of the possible space groups, including $P\bar{3}$, $P321$and $P\bar{3}m1$, was conducted, based on the absence of violation of extinction rules and the agreement between the model and data *F*(*hkl*) values ($R_{1}$ parameter). Specifically, the single-crystal goodness-of-fit parameters $R_{1}$, ${wR}_{2}$, and *GOF* (Goodness Of Fit) are defined as $R_{1}=\sum\left| |F_{O} \right|-\left| F_{c}| \right|/\sum\left| F_{O} \right|$, ${wR}_{2}=\{\sum{w\left( F_{O}^{2}-F_{c}^{2} \right)}^{2}/\sum{w\left( F_{O}^{2} \right)}^{2}{\}}^{\frac{1}{2}}, GOF =\left\{ \sum{w\left( F_{O}^{2}-F_{c}^{2} \right)}^{2} \right)/(n-p){\}}^{\frac{1}{2}}$, respectively. In these expressions, $F_{O}$ is the measured scattering amplitudes, $F_{c}$ is the calculated scattering amplitudes, $w$ is the weight, $n$ is the number of reflections, and $p$ is the total number of fitting parameters. For a good fit to the data, both the $R_{1}$ and residual charge should be as small as possible, and the *GOF* should approach 1. We show the results of $P\bar{3}$, $P321$, and $P\bar{3}m1$ in Tables S1, S2 and S3, respectively. Based on the $R_{1}$, residual charge, and *GOF* parameters (also summarized in Table 1 in the main text), $P\bar{3}$ structure offers the best fit for the data. The intensity distribution matches only the $P\bar{3}$ space group well.

**Table S1.** Structural Parameters for $P\bar{3}$ Space Group at 290 K^a)^

| **Atoms** | ***x*** | ***y*** | ***z*** | *Ueq* (Å^3^) × 10^3^ |
| --- | --- | --- | --- | --- |
| Rb | 0 | 0 | 0 | 26.1(2) |
| Fe | 0 | 0 | 1/2 | 15.6(2) |
| S1 | 2/3 | 1/3 | 0.29528(10) | 15.2(2) |
| O1 | 0.7118(4) | 0.6408(4) | 0.3560(2) | 20.2(3) |
| O2 | 2/3 | 1/3 | 0.1207(4) | 27.9(7) |
| Atom *U_11_*(Å^2^)×10^3^ *U_22_*(Å^2^)×10^3^ *U_33_*(Å^2^)×10^3^ *U_12_*(Å^2^)×10^3^ *U_13_*(Å^2^)×10^3^ *U_23_*(Å^2^)×10^3^  Rb 22.7(3) 22.7(3) 32.7(4) 0 0 11.36(13)  Fe 10.8(2) 10.8(2) 25.2(4) 0 0 5.42(12)  S 12.0(3) 12.0(3) 21.6(4) 0 0 6.00(13)  O1 16.1(7) 11.7(6) 32.9(7) -3.1(5) -4.2(5) 7.1(5)  O2 31.9(10) 31.9(10) 20.0(11) 0 0 16.0(5) | | | | |
|  | | | | |
| Space Group: $P\bar{3}$  *a* = 4.8468(12) Å, *c* = 8.266(3) Å, *D_x_* = 3.293 g/cm^3^  Measurement Temperature: 290 K  Wavelength: 0.41328 Å,  Absorption Coefficient: 2.331 mm^-1^  *F*(000) = 159.0  2*θ* Range : 2.87° to 40.2° -8≤ *h* ≤8, -5≤ *k* ≤ 5, and -13 ≤ *l* ≤13,  Number of Measured Reflections: 1746  Number of Independent Reflections: 510  Number of fitting parameters: 22  Restraints = 0  Max and Min Peak in Final Difference Map (Residual charge): 2.12/-1.26 e-/Å^3^  *R_1_* = 3.90 %, *wR_2_* = 10.7 %, Goodness of Fit = 1.076(*I* >= 2*σ* (*I*))  *R_1_* = 4.09 %, *wR_2_* = 10.8 % (all data) | | | | |

^a)^ Occupancy of Rb site = 0.870(4)

**Table S2.** Structural Parameters for $P321$ Space Group Refinement at 290 K^a)^

| **Atoms** | ***x*** | ***y*** | ***z*** | *Ueq* (Å^3^) × 10^3^ |
| --- | --- | --- | --- | --- |
| Rb | 0 | 0 | 1/2 | 23.6(8) |
| Fe | 0 | 0 | 0 | 14.0(7) |
| S1 | 2/3 | 1/3 | 0.7953(4) | 12.7(7) |
| O1 | 2/3 | 1/3 | 0.6215(14) | 29(3) |
| O2 | 0.3610(18) | 0.2850(20) | 0.8573(11) | 22(2) |
| Atom *U_11_*(Å^2^)×10^3^ *U_22_*(Å^2^)×10^3^ *U_33_*(Å^2^)×10^3^ *U_12_*(Å^2^)×10^3^ *U_13_*(Å^2^)×10^3^ *U_23_*(Å^2^)×10^3^  Rb 21(1) 21(1) 29(1) 0 0 10.5(5)  Fe 9.5(8) 9.5(8) 23(1) 0 0 4.8(4)  S 8.7(8) 8.7(8) 21(1) 0 0 4.4(4)  O1 33(4) 33(4) 20(4) 0 0 16(2)  O2 9(3) 15(3) 37(3) 3(3) 2(3) 3(2) | | | | |
|  | | | | |
| Space Group: $P321$  *a* = 4.8468(12) Å, *c* = 8.266(3) Å, *D_x_* = 3.293 g/cm^3^  Measurement Temperature: 290 K  Wavelength: 0.41328 Å,  Absorption Coefficient: 2.502 mm^-1^  *F*(000) = 159.0  2*θ* Range : 2.86° to 40.3° -8≤ *h* ≤8, -5≤ *k* ≤ 5, and -13 ≤ *l* ≤13,  Number of Measured Reflections: 1757  Number of Independent Reflections: 537  Number of fitting parameters: 23  Restraints = 0  Max and Min Peak in Final Difference Map (Residual): 3.29/-2.06 e-/Å^3^  *R_1_* = 8.65 %, *wR_2_* = 26.3 %, Goodness of Fit = 1.164 (*I* >= 2*σ* (*I*))  *R_1_* = 9.89 %, *wR_2_* = 25.6 % (all data) | | | | |

^a)^Occupancy of Rb site = 0.849(15)

**Table S3.** Structural Parameters for $P\bar{3}m1$Space Refinement at 290 K^a)^

| **Atoms** | ***x*** | ***y*** | ***z*** | *Ueq* (Å^3^) × 10^3^ |
| --- | --- | --- | --- | --- |
| Rb | 0 | 0 | 0 | 29(1) |
| Fe | 0 | 0 | 1/2 | 14(1) |
| S1 | 2/3 | 1/3 | 0.2950(4) | 13(1) |
| O1 | 2/3 | 1/3 | 0.120(2) | 26(3) |
| O2 | 0.928(2) | 0.639(2) | 0.353(1) | 49(2) |
| Atom *U_11_*(Å^2^)×10^3^ *U_22_*(Å^2^)×10^3^ *U_33_*(Å^2^)×10^3^ *U_12_*(Å^2^)×10^3^ *U_13_*(Å^2^)×10^3^ *U_23_*(Å^2^)×10^3^  Rb 25(1) 25(1) 37(2) 0 0 12(6)  Fe 9(1) 9(1) 22(1) 0 0 4.7(5)  S 9(1) 9((1) 20(1) 0 0 4.6(6)  O1 29(5) 29(5) 21(5) 0 0 15(2)  O2 32 (4) 32(4) 81(5) -4(3) 2(3) 15(3) | | | | |
| Space Group: $P\bar{3}m1$  *a* = 4.8468(12) Å, *c* = 8.266(3) Å, *D_x_* = 3.293 g/cm^3^  Measurement Temperature: 290 K  Wavelength: 0.41328 Å,  Absorption Coefficient: 2.316 mm^-1^  *F*(000) = 159.0  2*θ* Range : 2.86° to 40.3° -8≤ *h* ≤8, -5≤ *k* ≤ 5, and -13 ≤ *l* ≤13,  Number of Measured Reflections: 1757  Number of Independent Reflections:344  Number of fitting parameters: 22  Restraints = 0  Max and Min Peak in Final Difference Map (Residual): 4.93/-2.06 e-/Å^3^  *R_1_* = 11.5 %, *wR_2_* = 31.3 %, Goodness of Fit = 1.266 (*I* >= 2*σ* (*I*))  *R_1_* = 12.8 %, *wR_2_* = 31.9 % (all data) | | | | |

^a)^Occupancy of Rb site = 1.02(2)

3. FR domains and DFT-optimized crystal structures

The figure below depicts the connection between type-I and type-II FR domains and the DFT-optimized crystal structures for both RFSO and RbFe(MoO_4_)_2_ (RFMO), employing $P\bar{3}$ and $P\bar{3}m1$ models.


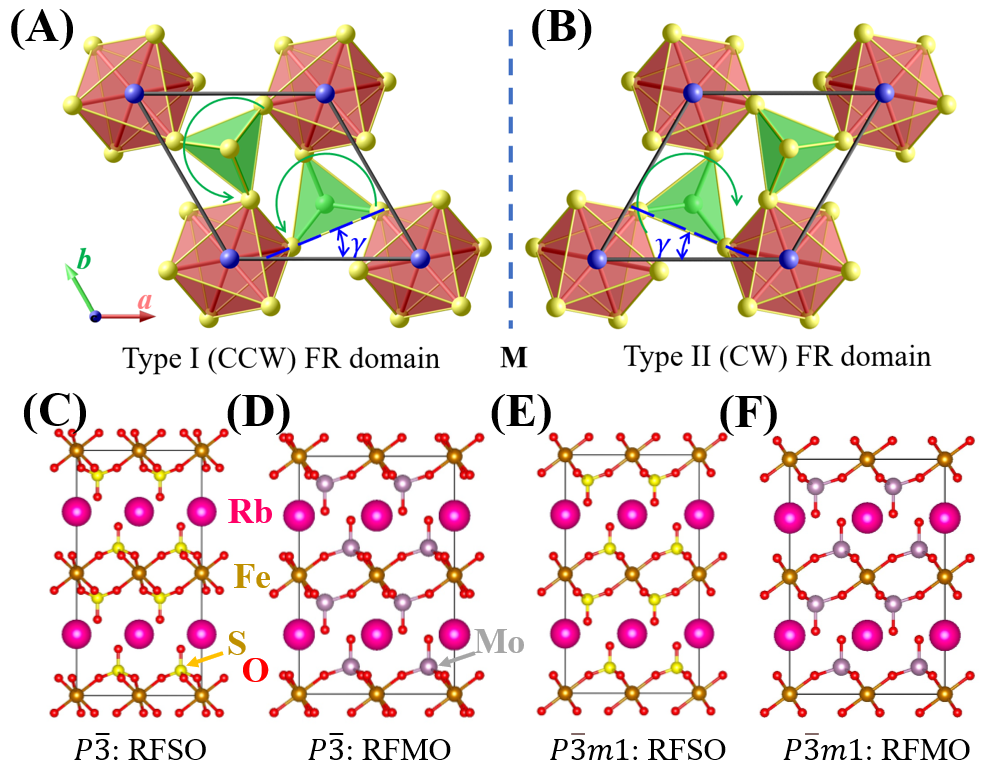


**Figure S2.** **(A)** The CCW rotational distortions (green circles with arrows) of SO_4_ and FeO_6_ for the type-I FR domain. The angle *γ* denotes the rotational distortion respective to the *a*-axis. **(B)** The CW rotational distortions (green circles with arrows) of SO_4_ and FeO_6_ for the type-II FR domain. The type-II FR domain is a mirror image of the type-I FR domain. The blue dashed line shows the mirror. **(C)** The DFT-optimized $P\bar{3}$ structure of RFSO. **(D)** The DFT-optimized $P\bar{3}$ structure of RFMO. **(E)** The DFT-optimized $P\bar{3}m1$ structure of RFSO. **(F)** The DFT-optimized $P\bar{3}m1$ structure of RFMO.

**4. Magnetic structure of RFSO.**

The representational analysis enables the determination of symmetry-allowed magnetic structures resulting from a second-order magnetic phase transition, considering the crystal structure before the transition and the propagation vector of the magnetic ordering.^[3-6]^ In our case, the crystal structure of RFSO before the phase transition is described in space group $P\bar{3}$ (#147). As depicted in Figure 2(A)-(B) in the main text, RFSO exhibits magnetic reflections at (1/3, 1/3, - 0.43), (1/3, 1/3, 0.43), and (1/3, 1/3, 0.57). All these magnetic reflections can be indexed by a magnetic propagation vector *k* ~ (1/3, 1/3, 0.43). The representational analysis was conducted using the program SARAh.^[4]^ The magnetic representation of the Fe site can be decomposed in terms of the irreducible representations (IRs) of *G_k_*:

$$\Gamma_{Mag}=\sum_{\nu} n_{\nu}\Gamma_{\nu}^{\mu}$$

where $n_{\nu}$ is the number of times that the IR $\Gamma_{\nu}$ of order *μ* appears in the magnetic representation $\Gamma_{Mag}$ for the chosen crystallographic site. The decomposition of the magnetic representation $\Gamma_{Mag}$ in terms of the non-zero IRs of *G_k_* for the magnetic Fe site examined, and their associated basis vectors, $\psi_{n}$, are given in Table S4. The labeling of the propagation vector and the IRs follows the scheme used by Kovalev.^[7]^

Only three IRs are possible for the given magnetic propagation vector and the lattice symmetry of RFSO. The magnetic structures corresponding to the IRs Γ_1_, Γ_2_, and Γ_3_ are shown in Figure S3(A), (B), and (C), respectively. Γ_1_ yields a spin-density wave magnetic structure with magnetic moments aligned along the *c*-axis, which is not chiral. Γ_2_ and Γ_3_ produce the left-handed and right-handed chiral helical magnetic structures, respectively. The chiral helical magnetic structure consists of a 120° configuration within each layer and a chiral helical twist between adjacent layers along the *c*-axis. Our polarized neutron diffraction results, as depicted in Figure 3 and Figure 4 in the main text, confirm the magnetic chirality in RFSO, thus ruling out Γ_1_. Furthermore, our polarized neutron diffraction results, shown in Figure 3 and Figure 4 in the main text, also demonstrate that the handedness of the magnetic structure (i.e., Γ_2_ or Γ_3_) can be controlled by the external electric field.

**Table S4.** Basis vectors for the space group $P\bar{3}$with *k* ~ (1/3, 1/3, 0.43). The decomposition of the magnetic representation for the Fe site (0, 0, 0.5) is $\Gamma_{Mag}=1\Gamma_{1}^{1}+1\Gamma_{2}^{1}+1\Gamma_{3}^{1}$.

**IR BV Atom BV components**

$m\parallel a$ $m\parallel b$ $m\parallel c$ $im\parallel a$ $im\parallel b$ $im\parallel c$

Γ_1_ $\psi_{1}$ Fe 0 0 3 0 0 0

Γ_2_ $\psi_{2}$ Fe 1.5 0 0 -0.866 -1.732 0

Γ_3_ $\psi_{3}$ Fe 1.5 0 0 -0.866 -1.732 0


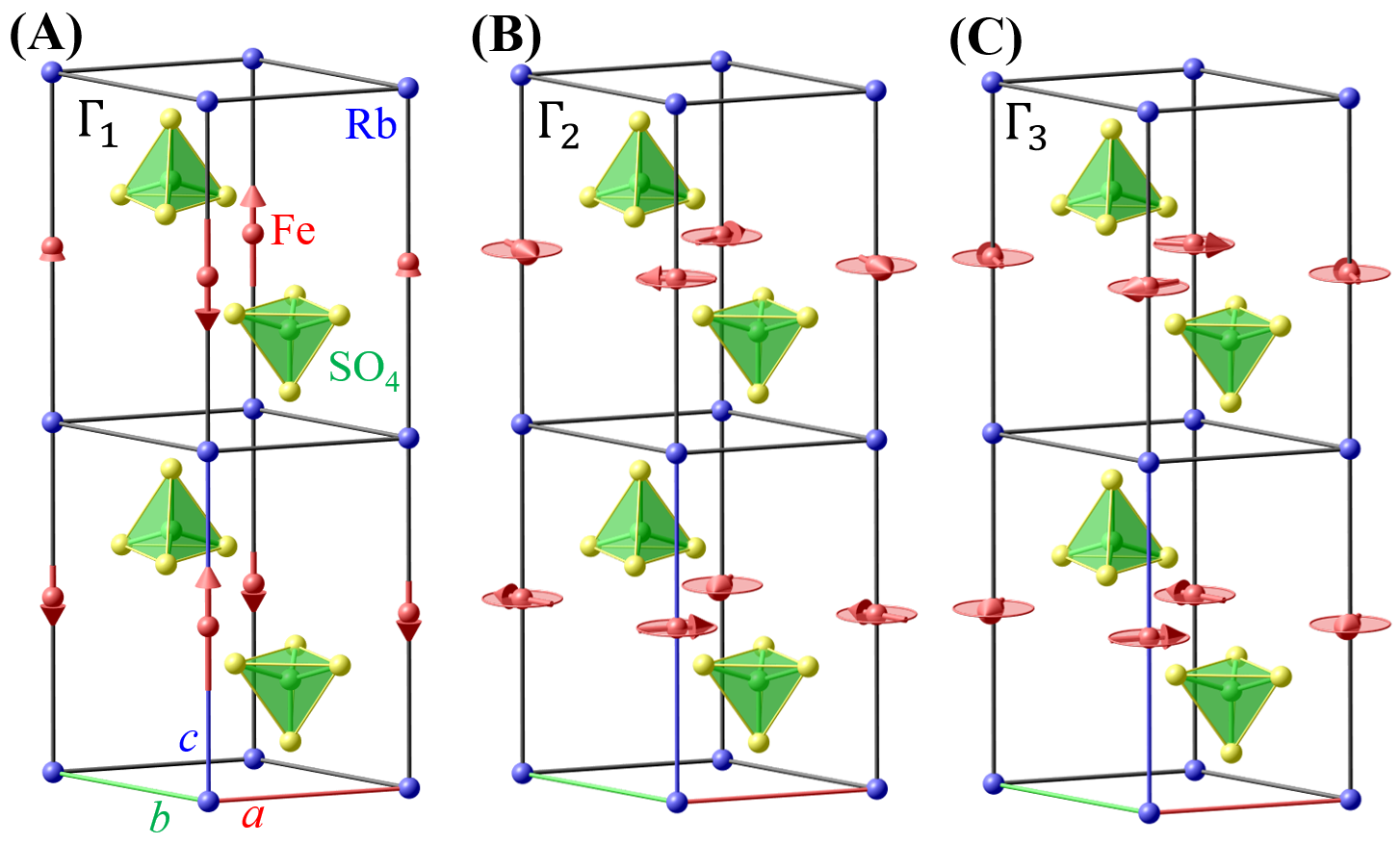


**Figure S3.** **(A)** The Γ_1_ magnetic structure shows the spin-density structure with magnetic moments aligned along the *c*-axis. **(B)** Γ_2_ and **(C)** Γ_3_ produce the left-handed and right-handed chiral helical magnetic structures, respectively.

**5. Additional neutron diffraction results**

Figure S4(A) shows the unpolarized neutron diffraction pattern observed at (2/3, 2/3, 0.5 ± Δ) for RFSO crystal #2. The intensity of the peak at (2/3, 2/3, 0.5 + Δ) is significantly greater than that at (2/3, 2/3, 0.5 - Δ), indicating the prevalence of one FR domain in the crystal. Figure S4(B) shows the polarized neutron diffraction pattern observed at (2/3, 2/3, - 0.43) at 1.6 K, with a zero (0 kV/cm) poling field. When the crystal remains unpoled, the $I_{x}^{\mp}$ and $I_{x}^{\pm}$ showcase nearly identical intensities, indicating the roughly equal distribution of left- and right-handed magnetic domains.


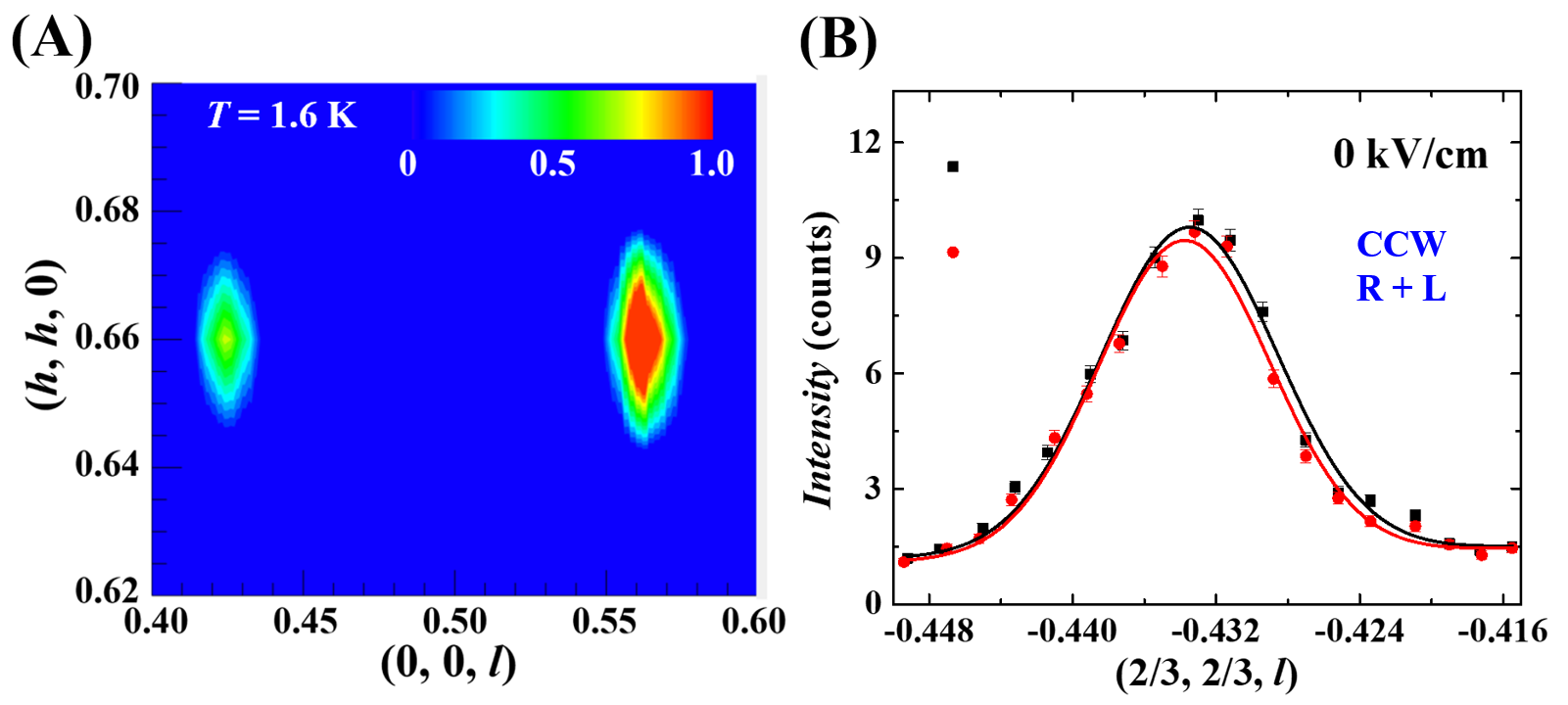


Figure S4. (A) Unpolarized neutron diffraction pattern in the (*h*, *h*, *l*) scattering plane measured at 1.6 K on an RFSO single crystal (#2). (B) Polarized neutron diffraction intensities of $\boldsymbol{I}_{\boldsymbol{x}}^{\boldsymbol{\pm}}$ and $\boldsymbol{I}_{\boldsymbol{x}}^{\boldsymbol{\mp}}$ measured at (2/3, 2/3, - 0.43) at 1.6 K, with a 0 kV/cm poling field. The CCW letter indicates the type of FR domain, and the R and L letters signify the handedness of the magnetic domain.

6. RA-SHG patterns

We conducted RA-SHG measurements at four locations on two distinct crystals (#3 and #4). In the case of crystal #3, the two positions are approximately 1 mm apart, while for crystal #4, the two positions are separated by approximately 2 mm. Remarkably, all four patterns exhibit identical characteristics, suggesting the presence of a homo-FR domain in RFSO crystals that extends for a distance of at least 2 mm. This domain size is approximately 50 times larger than that observed in RFMO, which is around 40 μm.


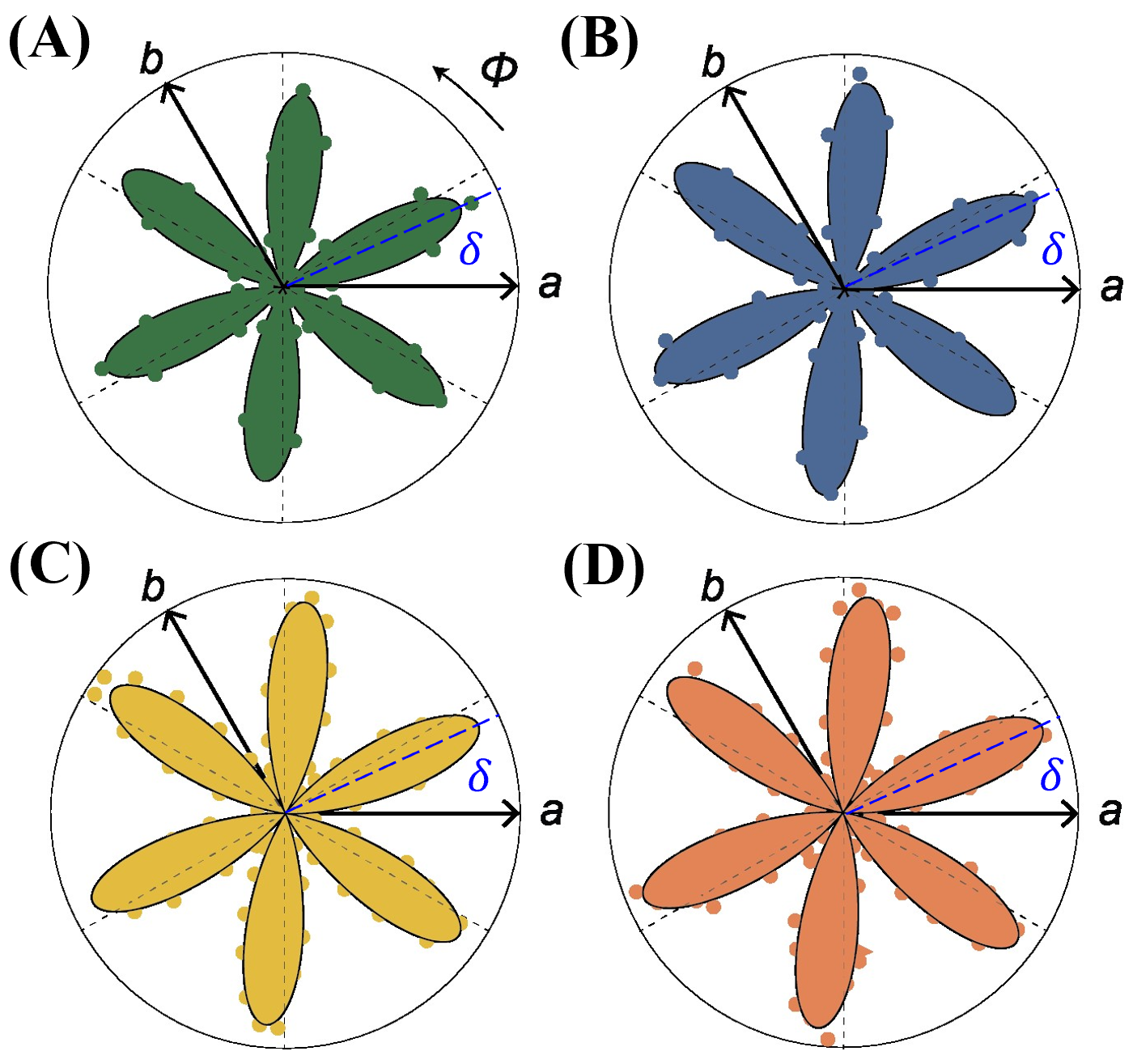


**Figure S5.** **(A)-(B)** RA-SHG patterns measured at two distinct regions (separated by approximately 1 mm) on RFSO crystal #3 in the parallel channel under normal incidence. The angle *δ* represents the rotational deviation of the patterns from the *a*-axis. **(C)-(D)** RA-SHG patterns measured at two different regions (separated by about 2 mm) on RFSO crystal #4.

References:

[1] Bruker (2016). APEX3, SAINT, and SADABS. Bruker AXS Inc., Madison, Wisconsin, USA.

[2] O. V. Dolomanov, L. J. Bourhis, R. J. Gildea, J. A. C. Howard, and H. Puschmann, *J. Appl. Cryst.* **2009**, 42, 339.

[3] A. S. Wills, *Phys. Rev. B.* **2001**, 63, 64430.

[4] A. S. Wills, *Physica B: Condensed Matter* **2000**, 276-278, 680-681.

[5] E. F. Bertaut, *Acta Crystallographica Section A* **1968**, 24, 217-231.

[6] E. F. Bertaut. *J. Magn. Magn. Mater.* **1981**, 24, 267.

[7] O. V. Kovalev, *Representations of the Crystallographic Space Groups* Edition 2 (Gordon and Breach Science Publishers, Switzerland, 1993).
